# Machine learning algorithms applied to the diagnosis of COVID-19 based on epidemiological, clinical, and laboratory data

**DOI:** 10.36416/1806-3756/e20240385

**Published:** 2025-03-18

**Authors:** Silvia Elaine Cardozo Macedo, Marina de Borba Oliveira Freire, Oscar Schmitt Kremer, Ricardo Bica Noal, Fabiano Sandrini Moraes, Mauro André Barbosa Cunha

**Affiliations:** 1. Faculdade de Medicina, Universidade Federal de Pelotas, Pelotas (RS) Brasil.; 2. Instituto Federal de Educação, Ciência e Tecnologia Sul-rio-grandense, Campus Pelotas, Pelotas (RS) Brasil.

**Keywords:** COVID-19/diagnosis, Artificial intelligence, Machine learning

## Abstract

**Objective:**

To predict COVID-19 in hospitalized patients with SARS in a city in southern Brazil by using machine learning algorithms.

**Methods:**

The study sample consisted of patients ≥ 18 years of age admitted to the emergency department with SARS and hospitalized in the Hospital Escola - Universidade Federal de Pelotas between March and December of 2020. Epidemiological, clinical, and laboratory data were processed by machine learning algorithms in order to identify patterns. Mean AUC values were calculated for each combination of model and oversampling/undersampling techniques during cross-validation.

**Results:**

Of a total of 100 hospitalized patients with SARS, 78 had information for RT-PCR testing for SARS-CoV-2 infection and were therefore included in the analysis. Most (58%) of the patients were female, and the mean age was 61.4 ± 15.8 years. Regarding the machine learning models, the random forest model had a slightly higher median performance when compared with the other models tested and was therefore adopted. The most important features to diagnose COVID-19 were leukocyte count, PaCO_2_, troponin levels, duration of symptoms in days, platelet count, multimorbidity, presence of band forms, urea levels, age, and D-dimer levels, with an AUC of 87%.

**Conclusions:**

Artificial intelligence techniques represent an efficient strategy to identify patients with high clinical suspicion, particularly in situations in which health care systems face intense strain, such as in the COVID-19 pandemic.

## INTRODUCTION

COVID-19 has been the most important health problem in the world since 2020. Following its emergence in December of 2019, in Wuhan, China, the disease spread quickly across the world and, in February of 2020, the WHO declared it a pandemic because of its global impact.^1^

There are currently around 750 million confirmed cases of COVID-19 and 7 million COVID-19-related deaths worldwide. In Brazil, the number of COVID-19 cases and deaths were extremely high during the pandemic, and the disease had a harmful impact on the public health system.^2^

Viral infections such as SARS-CoV-2 infection are dangerous because they spread very quickly; therefore, early detection and diagnosis have a positive impact on health strategies.^3^ Early in the COVID-19 pandemic, there were few diagnostic tests available in many countries, including Brazil; therefore, there was a need to select clinical and laboratory variables that could predict COVID-19 in order to proceed to nasal swab collection for RT-PCR to detect SARS-CoV-2 infection.^4^ Although COVID-19 mortality has declined, the existence of other circulating viruses makes it necessary to establish the correct diagnosis and reduce the risk of transmission. 

Artificial intelligence (AI) has been deployed at various levels of the health care system, including diagnosis,^5^ ^-^ ^7^ public health, clinical decision making, and therapeutics. Particularly, AI algorithms have been shown to be effective in improving the diagnosis and prognosis of COVID-19 through the creation of models including clinical and epidemiological characteristics, as well as biochemical data.^8^ ^-^ ^10^ The present study evaluated clinical and laboratory data to predict COVID-19 in hospitalized patients with SARS in a city in southern Brazil by using machine learning algorithms. 

## METHODS

The present study was conducted in the city of Pelotas, Brazil, which is the fourth most populated city in the state of Rio Grande do Sul, with a population of 325,685 inhabitants.^11^ The city of Pelotas is the largest of the 22 municipalities in the Third Regional Health District. Therefore, patients from some of the other municipalities are referred to health care facilities in Pelotas, and this was especially true during the COVID-19 pandemic. 

During the data collection period, the emergency department became the point of entry into the public health care system for patients from the city of Pelotas (and other municipalities) presenting with SARS. After undergoing an initial evaluation and RT-PCR for COVID-19, patients meeting the criteria for hospital admission were transferred to a public hospital able to receive them. In this context, the *Hospital Escola-Universidade Federal de Pelotas* became the most important center for receiving and treating patients with SARS during the COVID-19 pandemic. 

The study sample consisted of patients ≥ 18 years of age admitted to the emergency department with SARS and hospitalized in the *Hospital Escola - Universidade Federal de Pelotas* between March and December of 2020. Because the data were collected retrospectively, the requirement for written informed consent was waived. The study project was approved by the Brazilian National Research Ethics Committee (Protocol no. 37337720.2.0000.5317). 

We collected data on patient characteristics, including demographics (sex and age); comorbidities (e.g., obesity, diabetes, hypertension, cancer, and chronic respiratory disease); symptoms (e.g., cough, shortness of breath, chest pain, sore throat, and headache); vital signs (HR, RR, systolic blood pressure, diastolic blood pressure, and axillary temperature); and laboratory test results (e.g., hemoglobin level, leukocyte count, platelet count, and creatinine level). Table 1 shows the clinical and epidemiological characteristics of the patients suspected of having COVID-19. The missing values in the dataset were imputed by using the mean value of the features. The 100 rows were randomly divided into 70% for training and 30% for test. The continuous variables were normalized on the basis of the mean and standard deviation of the training sample. For the discrete variable, all values were in the interval between 0 and 1; therefore, no normalization was applied. 

**Table 1:** Epidemiological and clinical characteristics of patients suspected of having COVID 19.^a^

Variable	Original sample (n = 100)	Study sample (N = 78)
Sex, male	42 (42)	31 (39.7)
Age, years	61.4 ± 15.8	61.3 ± 15.4
Multimorbidity^b^	55 (58.5)	43 (57.3)
Hypertension	53 (54.6)	41 (53.2)
Diabetes	43 (44.3)	33 (42.9)
Obesity	13 (13.5)	11 (14.3)
Cancer	14 (14.6)	11 (14.3)
Chronic respiratory disease	18 (18.7)	15 (19.5)
Smoking	24 (25.5)	18 (24.0)
Days to onset of symptoms	9 [3-14]	9 [3-14]
Symptoms		
Cough	59 (59.0)	48 (61.5)
Shortness of breath	66 (66.0)	49 (62.8)
Chest pain	15 (15)	11 (14.1)
Sore throat	6 (6)	6 (7.7)
Runny nose or sneezing	10 (10)	9 (11.5)
Loss of smell or taste	6 (6)	5 (6.4)
Headache	11 (11.0)	10 (12.8)
Muscle or joint pain	43 (43)	34 (43.6)
Digestive symptoms	13 (13.0)	11 (14.1)
Fever	44 (44.0)	36 (46.1)
Vital signs		
HR, bpm	101 ± 19	100 ± 21
RR, cycles/min	24 ± 14	25 ± 16
Systolic blood pressure, mmHg	132 ± 25	130 ± 26
Diastolic blood pressure, mmHg	80 ± 15	80 ± 15
Axillary temperature, °C	36.7 ± 0.9	36.7 ± 0.9
Laboratory findings		
Hemoglobin, g/dL	12.5 ± 1.9	12.4 ± 2.0
Leucocytes, ×10^3^ cells/mm^3^	9.3 [6.8-13.7]	8.6 [6.5-13.7]
Band forms, %	4 [2-6]	4 [2-6]
Lymphocytes, %	13 [6-18]	13 [6-20]
Platelets, cells/µL	235,494 ± 100,953	240,787 ± 96,327
Creatinine, mg/dL	0.9 [0.7-1.2]	0.8 [0.7-1.1]
Troponin	10.8 [4.1-33.2]	9.7 [3.4-30.5]
D-dimer, mg/L	1.0 [0.7-1.6]	1.0 [0.7-1.5]
C-reactive protein, mg/L	98.2 [48.9-159.6]	80.7 [42.8-145.9]
ESR, mm	89 [52-126]	89 [51.5-122.5]
LDH, U/L	336 [280-441]	326 [269-441]

a Data presented as n (%), mean ± SD, or median [IQR]. ^b^Two or more of the following comorbidities: hypertension, diabetes, obesity, respiratory disease, smoking, cancer, HIV infection, and rheumatic disease.

Several machine learning algorithms were examined in the present study, including support vector machines, gradient boosting, multilayer perceptron (MLP), adaptive boosting, and decision trees. All these algorithms are known as supervised learning algorithms. In supervised learning, the model observes input-output pairs and the learning algorithm finds the optimal configuration of parameters resulting in a function that maps from input to output while minimizing a certain loss function.^12^ ^,^ ^13^

The choice to use several algorithms allows one to cover different methods, from more classic and simple statistical methods such as decision trees to ensemble learning, convex optimization, and gradient-based methods such as MLPs. Each approach has advantages and peculiarities that could or could not be suitable for the problem tackled in the present study. Therefore, a cross-validation step allowed us to verify which methods optimized a certain metric. 

In addition to the classification methods, because the collected dataset was imbalanced, techniques for oversampling were used in order to improve the overall performance of the system. Class imbalance poses serious problems for machine learning techniques. Some of the most conventional approaches to these problems are undersampling and oversampling. Oversampling consists in creating artificial data on the basis of the statistical behavior of the elements of the minority class, whereas undersampling consists in sampling the majority class in such a way that the dataset becomes balanced.^14^

Synthetic Minority Oversampling Technique (SMOTE) is one of the most notable oversampling methods available.^15^ SMOTE works by taking each sample in the minority class and creating synthetic samples in the lines that connect the sample with each *k*-nearest neighbors. Other oversampling methods include a variation of SMOTE, known as Borderline-SMOTE, and an adaptive synthetic sampling approach for imbalanced learning,^15^ ^-^ ^17^ both of which were tested in the present study. 

Each combination of model and sampling technique was optimized by using a grid search approach. In grid search, a list of possible values for each hyperparameter is created, and all combinations of between-values are examined. The cross-validation method used was k-fold, with k = 5. The programming language used was Python 3.6, and the following libraries were used: NumPy 1.19.5; imbalanced-learn 0.4.3; pandas 0.22.0; scikit-learn 0.21.0; SciPy 1.4.1; and statsmodels 0.9.0. 

After finding the best model, we were able to analyze other metrics, such as sensitivity, specificity, precision, and confusion matrix. We analyzed the relevance of the variables present in the data for the classification of the models. This allowed us to determine the importance of the variables used and the level of agreement between the model and previously established knowledge. 

## RESULTS

A total of 78 patients had information for RT-PCR testing for SARS-CoV-2 infection and were therefore included in the analysis. The study sample did not differ from the original sample (n = 100) with regard to the baseline characteristics (Table 1). The median value and interquartile range for each variable are shown in Table 1. Of the sample as a whole (N = 78), 42% were male, and the mean age was 61.4 ± 15.8 years. Nearly 60% of the study participants had two or more comorbidities, with hypertension and diabetes being the most prevalent (in 58.5% and 44.3%, respectively). One quarter of the study participants were current smokers. The median time elapsed since the onset of symptoms was 9 days (IQR: 3-14 days), the most common symptoms being shortness of breath (in 66%), cough (in 59%), fever (in 44%), and muscle or joint pain (in 43%). 

For a comprehensive analysis, each combination of classification model and oversampling/undersampling method was trained and cross-validated 30 times. This approach allowed the construction of a performance distribution for each pair. Given the imbalanced nature of the dataset, the performance was evaluated by means of the AUC metric. Models such as MLP, the random forest method, and gradient boosting achieved similar performance levels. Of those, the random forest model without any oversampling method showed the highest median performance, although it was only slightly higher than the median performance of the other models. Given that this combination not only yielded the best performance but also entailed lower computational costs than did the other two methods, it was selected for further analysis. 

**Figure 1:**
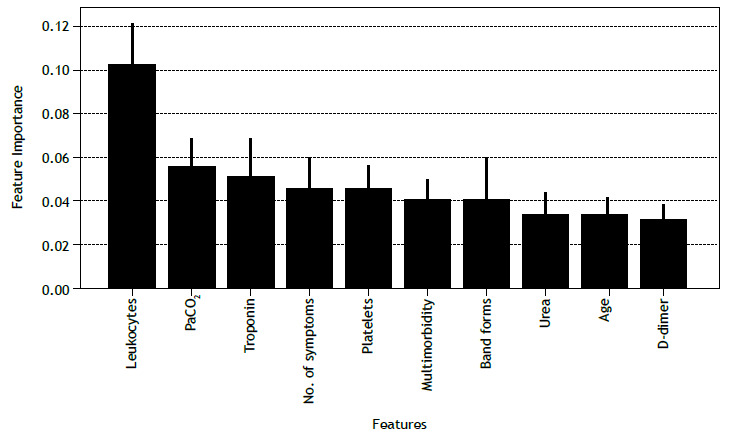
The ten most important features to diagnose COVID-19 with the use of a random forest algorithm.

The step of feature selection is also significantly important in the implementation of machine learning models. Additionally, when considering the practical aspects of implementing a process that will be directly dependent on data collection, it is useful to find the best trade-off between performance and number of features. First, to select which features can be useful for classification, one must have a measure of their importance in the overall performance of the algorithm. Different approaches can be used in order to extract feature importance in machine learning models, including permutation importance, SHAP, and mean decrease in impurity (MDI), the last being particularly suitable for models such as the random forest. MDI, also known as the Gini importance, explores the structure of the random forest to evaluate feature importance. Given that a random forest is an ensemble learning algorithm based on decision trees, MDI counts the times a feature is used to split a node in a tree, weighted by the number of samples it splits. This allows us to identify how relevant a certain feature is for generating a prediction. By evaluating the MDI for the trained model, we identified the ten most important features (Figure 1). 

**Figure 2:**
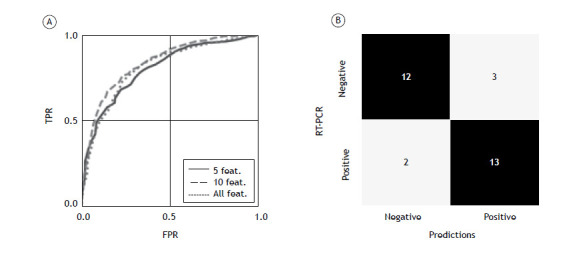
Random forest metrics. In A, ROC curves for different numbers of variables (all variables, five variables, and ten variables). In B, confusion matrix for the best model (i.e., the model including ten features). The ROC curve was plotted by averaging the ROC curves for 20 different trials, with random splits between training and test datasets. TPR: true-positive rate; and FPR: false-positive rate.

The process of analyzing feature relevance helps reduce computational cost, and, by reducing the number of features, it is possible to decrease the probability of introducing undesired bias due to the size of the training dataset. However, it is still important to evaluate the performance of the model with different numbers of features. As can be seen in Figure 2A, ROC curves were plotted for three different scenarios: all features; the five most relevant features; and the ten most relevant features. The best performance in terms of AUC was achieved by the model containing the ten most relevant features. As can be seen in Figure 2B, a confusion matrix of the model containing the ten most relevant features shows the relationship between the output of the model and the RT-PCR results, highlighting each type of correct and incorrect prediction. 

Table 2 presents key metrics that highlight the performance of the model across different feature sets. Notably, when the ten most relevant features were used, the performance of the model improved not only in terms of AUC (as can be seen in Figure 2A) but also in terms of sensitivity. It is important to emphasize that the output of the model can be interpreted similarly to a probability, which allows the definition of a threshold (a value between 0 and 1) to determine whether a numerical output results in a positive or negative result. This provides flexibility to balance between sensitivity and precision, thus reducing the occurrence of false positives and false negatives. For the results presented herein, a threshold of 0.5 was considered. 

**Table 2:** Key metrics of the random forest classifier used in the present study, by number of features included.^a^

Number of variables	Sensitivity	Precision	F1 score	AUC
All	0.714 [0.642-0.827]	0.742 [0.618-0.818]	0.717 [0.689-0.752]	0.824 [0.796-0.856]
5	0.721 [0.673-0.825]	0.659 [0.612-0.717]	0.695 [0.661-0.743]	0.795 [0.706-0.820]
10	0.757 [0.659-0.822]	0.746 [0.667-0.862]	0.75 [0.694-0.776]	0.867 [0.832-0.894]

a Data expressed as median [IQR].

## DISCUSSION

Since the WHO declared COVID-19 a pandemic on March 12, 2020, health care systems worldwide faced intense strain. This raised the need for exploring new and emerging technologies to meet the increasing health demand. One important challenge was the scarcity of medical supplies and diagnostic tools, especially in the first year of the pandemic. The limited availability of resources, including COVID-19 diagnostic tests, highlighted the need for developing tools to identify patients with high clinical suspicion of COVID-19. In this context, AI techniques represent an efficient strategy for detection, severity assessment, and therapeutic approach. 

In the last three years, many studies have investigated the role of imaging tests such as X-rays, CT scans, and ultrasound examination in the early diagnosis of COVID-19 through AI techniques.^18^ On the other hand, the integration of clinical data into AI algorithms has been less studied and could represent an effective strategy to face the challenges of COVID-19, particularly in scenarios in which imaging tests are not readily available. Previous studies evaluating the use of AI in COVID-19 diagnosis showed accuracy values of approximately 85%.^19^ Ahamad et al. reported that the most relevant predictive symptoms were fever (41.1%), cough (30.3%), lung infection (13.1%), and runny nose (8.43%).^20^ Similarly, we found an accuracy of 84% when we included ten variables in the model. However, the most relevant predictors in our study were leukocyte count, PaCO_2_, troponin levels, duration of symptoms in days, platelet count, multimorbidity, presence of band forms, urea levels, age, and D-dimer levels. In this context, Silveira found an association between blood count and COVID-19 diagnosis through a gradient boosting model, with an accuracy of 80.0%, a sensitivity of 75.6%, and a specificity of 82.0%.^21^ The variables that had the greatest influence on the predictive decision were basophil count, eosinophil count, and leukocyte count.^21^ It is important to highlight that our objective was to predict the probability of a COVID-19 diagnosis in patients hospitalized with SARS. 

One of the main limitations of the present study is the relatively small number of samples, especially in comparison with most machine learning applications.^18^ ^-^ ^20^ However, despite this limitation, the achieved performance demonstrates the value of the method as a useful tool for the health care system. To enhance the performance of the model, it is crucial to expand data collection to different municipalities. This would not only increase the size of the dataset but also improve the generalization capability of the model. Additionally, continuous retraining of the model would enable it to adapt to the evolving effects of the virus on the population. Such efforts would not only increase the impact of the model but also provide a deeper understanding of the long-term effects and behavior of the COVID-19 pandemic. 

Although AI-based tools do not replace medical evaluation, their contribution is unequivocal in improving the management of several issues and health problems. Particularly in pandemic situations, AI-based tools can help to make rapid decisions related to the diagnosis and prevention of disease spreading. Thus, given that in the future the health care system might be faced with other pandemics, there is a need for continued improvement of AI technologies. Future studies should focus on strengthening current technologies to detect, monitor, and diagnose emerging and potentially life-threatening medical conditions. 
